# Efficacy and safety of cadonilimab combined with AG chemotherapy in patients with unresectable locally advanced or metastatic pancreatic ductal adenocarcinoma: a retrospective real-world study

**DOI:** 10.3389/fimmu.2025.1654425

**Published:** 2025-10-09

**Authors:** Wenke Qin, Yan Du, Xuean Zhao, Yongqing Zhao, Yan Zhang, Shuze Zhang, Zengxi Yang, Xin Li, Jubao Niu, Dewen Zhao, Kongyuan Wei, Hui Zhang

**Affiliations:** ^1^ The Second School of Clinical Medicine, Lanzhou University, Lanzhou, China; ^2^ Department of General Surgery, The Second Hospital & Clinical Medical School, Lanzhou University, Lanzhou, China; ^3^ Department of General Surgery, Jingtai County Hospital of Traditional Chinese Medicine, Baiyin, China; ^4^ Department of Hepatobiliary Surgery, The First Affiliated Hospital of Xi’an Jiao Tong University, Xi’an Jiao Tong University, Xi’an, China

**Keywords:** cadonilimab, pancreatic neoplasms, CTLA-4 Antigen, Ca19-9 antigen, bispecific

## Abstract

**Background:**

At the time of diagnosis, 80% of patients with pancreatic ductal adenocarcinoma (PDAC) are already at an unresectable advanced stage. The median overall survival (mOS) with traditional AG chemotherapy is only 8.5 months. The bispecific antibody Cadonilimab targeting PD-1 and CTLA-4 has shown immuno-oncological synergy in solid tumors, but evidence in PDAC is limited.

**Methods:**

This study is a single-center retrospective study that included 14 patients with advanced pancreatic ductal adenocarcinoma. These patients received Cadonilimab (10 mg/kg, Q3W) in combination with AG chemotherapy. The primary endpoint was the disease control rate (DCR, RECIST 1.1).

**Results:**

The disease control rate (DCR) reached 85.7%, and the objective response rate (ORR) was 14.3%; the median progression-free survival (mPFS) was 7.87 months, and the median overall survival (mOS) was 11.45 months. Among patients with a decrease in CA19-9, the median overall survival was significantly prolonged (12.92 vs 8.89 months, P = 0.027); patients with a platelet-lymphocyte ratio (PLR) ≤ 165.62 had a significantly better mOS (12.37 vs 8.74 months, P = 0.025). Although the incidence of grade 3 treatment-related adverse events was 57.1%, no grade 4 treatment-related toxic events were observed.

**Conclusion:**

In conclusion, cadonilimab combined with AG chemotherapy demonstrated promising antitumor activity and manageable safety in PDAC. Dynamic monitoring of PLR and CA19–9 may aid efficacy evaluation, but these exploratory findings require confirmation in prospective multicenter trials.

## 1 Introduction

Pancreatic ductal adenocarcinoma (PDAC), as one of the most aggressive malignant tumors in the digestive system, has a continuously rising incidence and mortality rate. According to the latest statistics from the American Cancer Society, it is estimated that there will be 67,440 new cases of PDAC and 51,980 deaths in 2025, making it the third leading cause of cancer-related death, with a 5-year relative survival rate of only 13% (1). Currently, first-line treatment for advanced PDAC mainly consists of albumin-bound paclitaxel combined with gemcitabine (AG regimen) or FOLFIRINOX (fluorouracil, leucovorin, irinotecan, and oxaliplatin), but there are significant bottlenecks in efficacy: the median overall survival (mOS) for the AG regimen is 8.5 months, while for the FOLFIRINOX regimen it is 11.1 months, and over 80% of patients experience disease progression within 6–9 months after treatment (2, 3). Although immune checkpoint inhibitors such as PD-1/CTLA-4 have made breakthroughs in multiple cancer types, PDAC has not been able to overcome treatment challenges due to its highly immunosuppressive tumor microenvironment and inherent chemotherapy resistance. Based on this, exploring the synergistic enhancement strategy of AG chemotherapy combined with novel bispecific antibodies (such as the PD-1/CTLA-4 bispecific antibody Cadonilimab) has become an important direction to overcome treatment resistance in PDAC.

Immune checkpoint inhibitors (ICIs) have significantly improved patient survival in solid tumors such as melanoma and non-small cell lung cancer (4, 5). However, their monotherapy efficacy in PDAC remains difficult to overcome. Studies have shown that PDAC has a highly immunosuppressive microenvironment (rich in fibrous stroma, regulatory T cells [Tregs], and myeloid-derived suppressor cells) and multiple immune evasion mechanisms (such as antigen presentation defects and overexpression of co-inhibitory molecules), which are the core reasons for the failure of ICIs (4, 6, 7). Given that chemotherapy drugs have immune regulatory potential (such as inducing immunogenic cell death and enhancing tumor antigen release), researchers have attempted to combine ICIs with the AG regimen to achieve synergistic effects (8). Preliminary clinical data indicate that the PD-1 inhibitor pembrolizumab combined with the AG regimen can increase the median progression-free survival (mPFS) and overall survival (mOS) in metastatic PDAC to 9.1 months and 15.0 months, respectively (9). However, the PD-1 monoclonal antibody nivolumab combined with the AG regimen failed to surpass the efficacy of traditional chemotherapy (10), suggesting that blocking the PD-1 pathway alone may be insufficient to reverse the immune resistance of PDAC (6).

Cytotoxic T lymphocyte-associated antigen 4 (CTLA-4), as an early T cell activation negative regulator, forms a complementary immunosuppressive mechanism with the PD-1 pathway: CTLA-4 mainly inhibits T cell activation in lymph nodes, while PD-1 weakens the effector function of T cells in peripheral tissues (11, 12). Preclinical evidence suggests that dual pathway blockade can significantly enhance anti-tumor activity by promoting T cell clonal proliferation, reducing the proportion of regulatory T cells, and remodeling the tumor microenvironment (13). In solid tumors such as melanoma and hepatocellular carcinoma, PD-1/CTLA-4 dual antibody combination regimens have shown survival benefits superior to monotherapy (14–16). However, PDAC-related research still faces challenges, such as the CCTG PA.7 phase II trial, where the PD-L1/CTLA-4 dual inhibitor (Durvalumab + Tremelimumab) combined with the AG regimen failed to improve patient survival and was associated with an increased incidence of grade 3 or higher toxicities (47% vs 26%) (17), indicating an urgent need for innovative improvements to this treatment regimen. Despite encouraging signals from early-phase studies, PD-1–based regimens have not altered the therapeutic landscape of PDAC. Pembrolizumab plus gemcitabine/nab-paclitaxel achieved mPFS 9.1 and mOS 15.0 months, but the small, single-arm design precluded firm conclusions (9). A phase I trial of nivolumab plus chemotherapy similarly failed to exceed historical benchmarks (10), and the randomized CCTG PA.7 trial of durvalumab plus tremelimumab did not improve OS or PFS while increasing grade ≥3 toxicities (17). Cadonilimab, a tetravalent PD-1/CTLA-4 bispecific with an Fc-silent, pH-dependent design, enables high-avidity intratumoral engagement and has shown a lower incidence of high-grade irAEs than conventional doublets (18–20). On this basis, we hypothesized that cadonilimab plus AG could overcome PDAC’s immune-refractory microenvironment and warranted real-world evaluation.

Cadonilimab is a tetravalent bispecific antibody targeting PD-1 and CTLA-4, which binds to PD-1 (KD = 1.33 nM) and CTLA-4 (KD = 3.79 nM) with high affinity through steric hindrance effects, forming an “immune synergistic activation domain” locally in tumors, significantly enhancing CD8+ T cell infiltration and interferon-γ secretion (18, 21). Its unique structural design (Fc silent mutation and pH-dependent binding) can reduce peripheral tissue-targeted toxicity, and clinical data show that the incidence of immune-related adverse events (irAEs) (32.1%) is significantly lower than that of traditional dual drug combinations (≥60%) (22, 23). In recurrent/metastatic cervical cancer (23), unresectable hepatocellular carcinoma (aHCC), and advanced gastric cancer (G/GEJ), Cadonilimab monotherapy or combination therapy has demonstrated significant survival benefits (mOS reaching 17.5-22.1 months) (20, 21, 24), but its application in PDAC has not been reported.

Based on the potential synergistic mechanism of PD-1/CTLA-4 dual pathway blockade and chemotherapy, this study aims to systematically evaluate the treatment response and safety profile of Cadonilimab combined with albumin-bound paclitaxel/gemcitabine (AG) regimen in patients with unresectable locally advanced or metastatic pancreatic ductal adenocarcinoma (PDAC) through a single-center retrospective study. The core hypothesis is that PD-1/CTLA-4 dual blockade can enhance tumor antigen-specific T cell responses and inhibit regulatory T cell (Tregs) function, synergizing with the immunomodulatory effects of AG chemotherapy to overcome PDAC treatment resistance (25–27). By assessing the dynamic changes of CA19–9 and baseline inflammatory markers, the study will explore their correlation with clinical outcomes (ORR, PFS, OS) and screen for prognostic biomarkers (28, 29).

## 2 Materials and methods

### 2.1 Study design and patient population

This study is a single-center retrospective study that included patients with unresectable locally advanced or metastatic pancreatic ductal adenocarcinoma (PDAC) who received Cadonilimab (10 mg/kg IV Q3W) combined with the AG regimen (albumin-bound paclitaxel + gemcitabine, D1/8 IV Q3W) at Lanzhou University Second Hospital from January 3, 2023, to April 12, 2024.Cadonilimab was used based on its efficacy in other solid tumors (21, 23, 24) and MDT consensus for patients with limited treatment options.Inclusion criteria included: histologically confirmed PDAC assessed as unresectable by a multidisciplinary team (MDT) (based on the NCCN pancreatic cancer guidelines v2.2021 unresectable criteria (30)); at least one measurable lesion according to RECIST v1.1 standards (31); age 18–75 years, ECOG PS 0-1 (32), expected survival ≥3 months (3); completion of ≥1 cycle of combination therapy with a complete efficacy assessment record. Exclusion criteria: previous treatment with PD-1/CTLA-4 inhibitors; presence of other malignancies or severe autoimmune diseases; bone marrow suppression: grade 3 or higher hematological toxicity (CTCAE v5.0 (33)): absolute neutrophil count (ANC) <1.0×10^9^/L; platelet count (PLT) <50×10^9^/L; hemoglobin (Hb) <8.0 g/dL; liver and kidney dysfunction: liver function Child-Pugh B/C grade or abnormal laboratory indicators: total bilirubin (TBIL) >1.5× upper limit of normal (ULN); ALT/AST >3×ULN (for liver metastasis patients >5×ULN); renal dysfunction: estimated glomerular filtration rate (eGFR) <45 mL/min/1.73m² (CKD-EPI formula). A total of 14 patients were finally included, and this study is a retrospective analysis, with ethical approval (approval number: 2025A-558) and informed consent waived after data collection was completed.This retrospective study was conducted to generate preliminary efficacy and safety data, which is a prerequisite for applying for subsequent prospective trials as required by the Ethics Committee of the Second Hospital & Clinical Medical School, Lanzhou University. Such a stepwise approach ensures that innovative therapeutic strategies are evaluated in a controlled manner before large-scale validation.

### 2.2 Endpoints and assessments

The primary endpoint of this study is the disease control rate (DCR, CR+PR+SD), defined according to the RECIST v1.1 standard as the proportion of patients with complete response (CR), partial response (PR), and stable disease (SD); secondary endpoints include objective response rate (ORR), progression-free survival (PFS, from the start of treatment to radiographic progression/death), overall survival (OS, from the start of treatment to all-cause mortality), and safety (adverse events graded by CTCAE v5.0 (33)). Imaging assessments are performed using enhanced CT (1 mm slice thickness, iodine contrast agent 350 mgI/mL), interpreted blindly by two independent radiologists for baseline and changes in lesions after every 3 cycles of treatment, with disputed results arbitrated by a third senior physician; laboratory monitoring includes peripheral blood inflammatory markers within 24 hours before treatment and on day 1 of each cycle, including: absolute neutrophil count (ANC), platelet count (PLT), absolute lymphocyte count (ALC), absolute monocyte count (AMC), and their composite parameters—neutrophil-lymphocyte ratio (NLR), platelet-lymphocyte ratio (PLR), lymphocyte-monocyte ratio (LMR), serum CA-199 (Roche electrochemiluminescence method, a decrease of ≥50% after 8 weeks of treatment defined as biochemical response), and lactate dehydrogenase (LDH, enzyme rate method, fluctuations >20% considered significant changes). Serum CA19–9 analysis was performed in all enrolled patients, with the aim of establishing an efficacy evaluation system based on the trajectory of this biomarker’s concentration changes.

Given PDAC’s desmoplastic biology (34, 35) and the possibility of delayed or non-conventional responses with immunotherapy, we prespecified disease control rate (DCR = CR+PR+SD per RECIST v1.1) as the primary endpoint to capture clinically meaningful stabilization together with responses. Objective response rate (ORR), progression-free survival (PFS), and overall survival (OS) were defined *a priori* as key secondary endpoints. This choice aligns with RECIST v1.1 (31) definitions and iRECIST (36) considerations for immune-related response kinetics.

### 2.3 Statistical analysis

**Figure 1:**
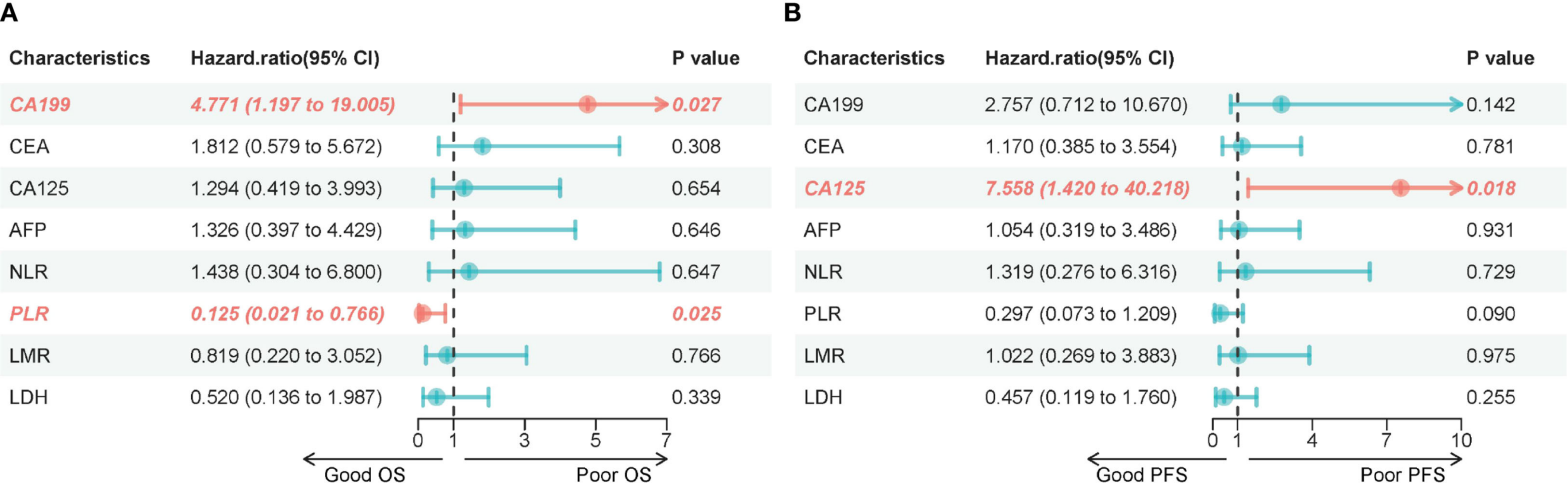
**(A)** Forest plot of univariable hazard ratios for overall survival (OS). Points represent HRs and horizontal bars the 95% CIs from Cox models for each baseline variable; the dashed vertical line marks HR = 1. **(B)** Forest plot of univariable hazard ratios for progression-free survival (PFS). Points represent HRs and horizontal bars the 95% CIs from Cox models for each baseline variable; the dashed vertical line marks HR = 1.

In the intention-to-treat population, an analysis of efficacy and safety outcomes was performed for patients who underwent one or more treatments followed by scans. Categorical variables were described using frequencies and percentages, while continuous variables were described using means and standard deviations, as well as medians, maximums, and minimums. Fisher’s exact test was used to assess response differences between clinical subgroups and other binary outcomes. The log-rank test compared survival functions between different subgroups. The optimal cutoff values for PLR, NLR, LMR, and LDH were calculated using the R Foundation (165.63, 3.03, 2.26, and 257.00). All statistical analyses and visualizations were conducted using R language (4.2.2). Forest-plot rendering of univariable effects. For overall survival (OS) and progression-free survival (PFS), we fitted univariable Cox proportional-hazards models for each baseline clinical/laboratory variable and reported hazard ratios (HRs) with 95% CIs. Effect sizes were visualized using forest plots (**Figure 1A** for OS; **Figure 1B** for PFS); the vertical reference line denotes HR = 1. Model proportionality and data completeness checks are summarized in **Supplementary Methods**. A p-value of less than 0.05 was considered statistically significant.The optimal cutoff values for PLR (165.63), NLR (3.03), LMR (2.26), and LDH (257.00), derived from limited data, may carry overfitting risk, and their generalizability requires further validation in larger cohorts.

Given the retrospective single-center design and small sample size (n=14), all analyses were prespecified as exploratory. Using Freedman’s approximation for time-to-event endpoints at a two-sided α=0.05, the minimum detectable hazard ratio (HR) with ~10–12 observed deaths is approximately 0.41–0.45 (37, 38). Our observed OS signal (median 11.45 vs historical 8.5 months; HR ≈0.74) lies outside this detectable range, implying a substantial risk of type II error (false-negative). The type I error was controlled at α=0.05 by design. For the primary binary endpoint, DCR (85.7%, 12/14 patients) yielded a 95% CI of ≈60.1%–96.0%, underscoring imprecision (39). A summary of type I/II error estimates is provided in **Supplementary Table 1**.

## 3 Results

### 3.1 Baseline demographic and clinical characteristics

A total of 14 patients with unresectable locally advanced/metastatic pancreatic ductal adenocarcinoma (PDAC) were included. The baseline characteristics are shown in **Table 1**, and the blood indicators are shown in **Tables 2** and **3**. The median age was 58.5 years (range 41–75 years), with a male-to-female ratio of 1:1. Disease staging: 2 cases of locally advanced (14.3%), 7 cases of distant metastasis (50.0%), and 5 cases of locally advanced with metastasis (35.7%). Tumor location: 6 cases in the head of the pancreas (42.9%), 5 cases in the body (35.7%), and 3 cases in the tail (21.4%). ECOG PS scores: 4 cases with a score of 0 (28.6%), and 10 cases with a score of 1 (71.4%).

**Table 1:** Baseline patient and demographic and disease characteristics.

Variable	N = 14
Gender, n (%)	
Female	7.0 (50.0%)
Male	7.0 (50.0%)
Age	
Mean (SD)	58.5 (10.1)
Median [Min, Max]	56.5 [41.0, 75.0]
Location, n (%)	
Body	5.0 (35.7%)
Head	6.0 (42.9%)
Tail	3.0 (21.4%)
Stage, n (%)	
Locally advanced	2.0 (14.3%)
Distant metastasis	7.0 (50.0%)
All	5.0 (35.7%)
BMI	
Mean (SD)	22.3 (2.9)
Median [Min, Max]	21.7 [18.3, 28.0]
ECOGPS, n (%)	
0	4.0 (28.6%)
1	10.0 (71.4%)
Treatment cycle	
Mean (SD)	4.2 (3.0)
Median [Min, Max]	3.5 [1.0, 12.0]

**Table 2:** The dynamics of tumor markers.

Variable	N = 14
CA199, n (%)	
Decrease	6.0 (42.9%)
Increase	8.0 (57.1%)
CEA, n (%)	
Decrease	8.0 (57.1%)
Increase	6.0 (42.9%)
CA125, n (%)	
Decrease	9.0 (64.3%)
Increase	5.0 (35.7%)
AFP, n (%)	
Decrease	10.0 (71.4%)
Increase	4.0 (28.6%)

**Table 3:** The dynamics of inflammation-related hematological indices.

Variable	N = 14
NLR	
Mean (SD)	3.9 (4.8)
Median [Min, Max]	2.4 [0.9, 19.7]
PLR	
Mean (SD)	132.3 (94.7)
Median [Min, Max]	128.4 [1.3, 405.0]
LMR	
Mean (SD)	3.4 (1.8)
Median [Min, Max]	2.9 [0.3, 6.4]
LDH	
Mean (SD)	196.7 (83.3)
Median [Min, Max]	200.5 [0.4, 307.0]

### 3.2 Efficacy

**Figure 2:**
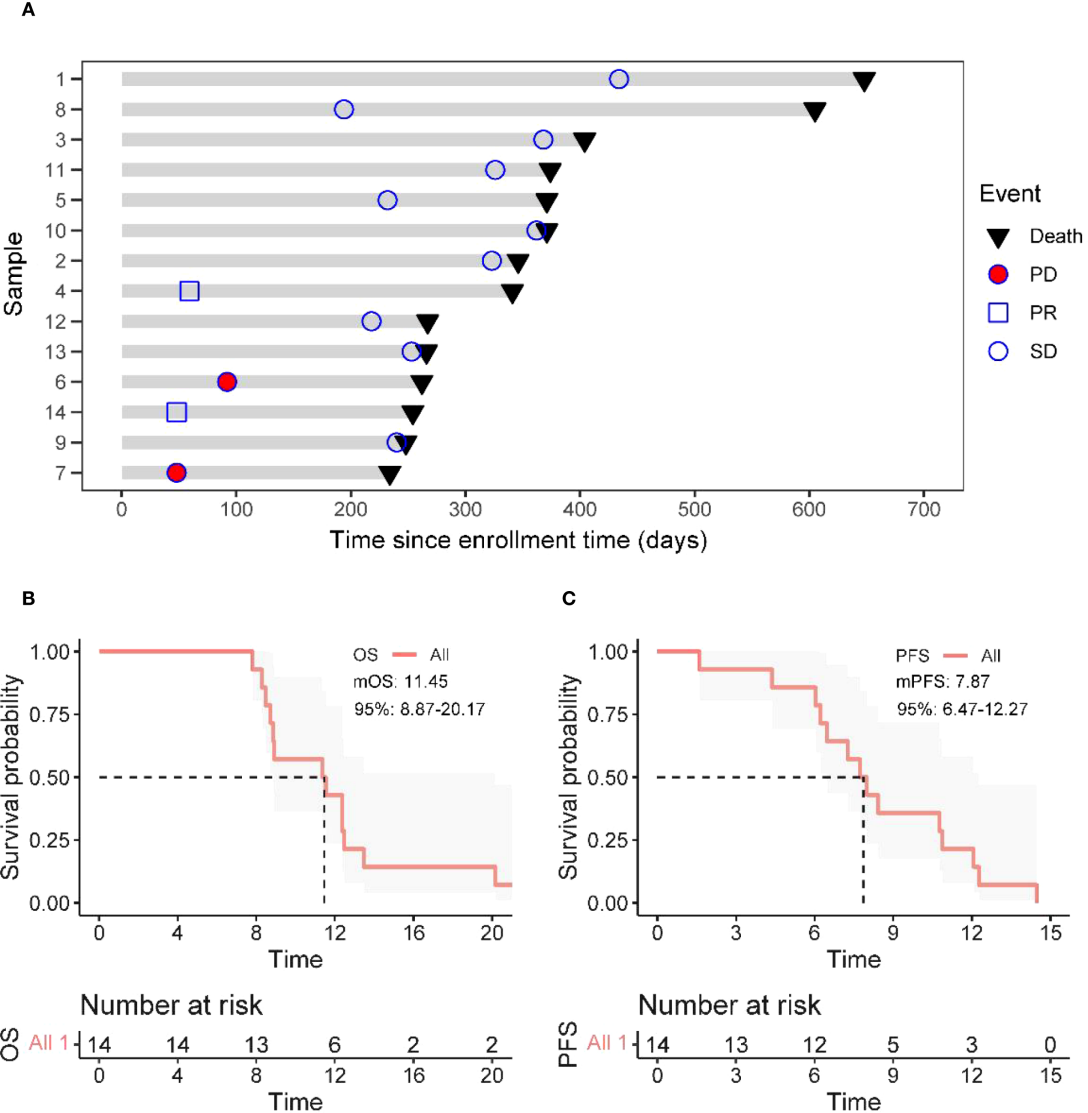
Treatment response and survival analysis. **(A)** Duration of responses of patients in the ITT (Intent-to-Treat) population. Each bar length represents each patient’s treatment duration. **(B, C)** The Kaplan–Meier curves of OS (Overall Survival) and PFS (Progression - Free Survival) in all enrolled patients.

As of the data cutoff in May 2024, this study included a total of 14 patients. After three cycles of treatment, the assessment showed: partial response (PR) in 2 cases (14.3%), stable disease (SD) in 10 cases (71.4%), and disease progression (PD) in 2 cases (14.3%), with an objective response rate (ORR) of 14.3% and a disease control rate (DCR) of 85.7% (**Table 4**). Survival analysis indicated a median progression-free survival (mPFS) of 7.87 months (95% CI 6.47-12.27) and a median overall survival (mOS) of 11.45 months (95% CI 8.87-20.17). The Kaplan-Meier curves for OS and PFS are shown in **Figures 2B, C**, and the efficacy dynamic swimlane diagram is shown in **Figure 2A**. To facilitate interpretation, we generated forest plots of univariable HRs for OS and PFS (**Figures 1A, B**). The plots highlight variables associated with favorable outcomes (e.g., PLR ≤ 165.6, on-treatment CA19–9 decline), whereas other covariates did not reach statistical significance (95% CI crossing HR = 1).

**Table 4:** Tumor responses.

Tumor response, n (%)	All (n =14)
Complete response	0(0.0)
Partial response (PR)	2 (14.3)
Stable disease (SD)	10 (71.4)
Progressive disease (PD)	2 (14.3)
ORR (CR + PR)	2 (14.3)
DCR (CR + PR + SD)	12 (85.7)

DCR, disease control rate; ORR, objective response rate.

### 3.3 Safety

All patients (100.0%) experienced treatment-related adverse events (TRAE), with a grade 3 TRAE incidence of 57.1% (8/14), primarily consisting of nausea (21.4%, 3/14) and decreased appetite (14.3%, 2/14), with no grade 4 or higher TRAE observed.Given symptom overlap, GI events were adjudicated as chemotherapy-consistent or indeterminate, and no immune-consistent colitis was identified. This pattern is coherent with the AG regimen’s known GI profile (2)and with cadonilimab clinical data showing uncommon GI-type irAEs and grade ≥3 events mainly hematologic (19, 20). Common TRAEs (incidence >50%) included decreased appetite (78.6%), weight loss (71.4%), nausea (71.4%), and fatigue (64.3%) (**Table 5**). No immune-related adverse events (irAE) were reported in the entire group.Grade 3 TRAEs (57.1%) were mainly gastrointestinal, comparable to FOLFIRINOX (54%) (3)and lower than traditional PD-1/CTLA-4 dual regimens (≥60%) (17), likely due to Cadonilimab’s Fc-silent design.Adverse events (AEs) were recorded at each cycle and coded to MedDRA. Severity was graded independently by two investigators per CTCAE v5.0 (41), with discrepancies resolved by a senior adjudicator. Because nonspecific gastrointestinal symptoms are common with nab-paclitaxel plus gemcitabine (AG) and may also occur during immunotherapy, attribution was prespecified as chemotherapy-consistent, immune-consistent, or indeterminate, adjudicated while blinded to biomarker data. This framework acknowledges uncertainty, aligns with the known AG toxicity profile (2), and is consistent with reports of predominantly hematologic AEs and low rates of immune-mediated colitis in cadonilimab trials (19, 20). Guidance for immune-related AE management followed established consensus recommendations (42, 43).

**Table 5:** Treatment related adverse events (TRAE) ≥10%.

Effects	All grades	Grade ≥3
At least one TRAE	14(100)	8(57.14)
Alanine aminotransferase increased	6(42.86)	0(0.0)
Aspartate aminotransferase increased	7(50.00)	0(0.0)
Thrombocytopenia	2(14.29)	0(0.0)
Leukocytopenia	8(57.14)	0(0.0)
Neutropenia	8(57.14)	0(0.0)
Diarrhea	2(14.29)	1(7.14)
Fatigue	9(64.29)	0(0.0)
Constipation	2(14.29)	0(0.0)
Hair loss	5(35.71)	0(0.0)
Hyperbilirubinemia	2(14.29)	0(0.0)
Hypertension	3(21.43)	0(0.0)
Hypothyroidism	2(14.29)	0(0.0)
Nausea	10(71.43)	3(21.43)
Hypoalbuminemia	7(50.00)	1(7.14)
Loss of appetite	11(78.57)	2(14.29)
Rash	2(14.29)	0(0.0)
Weight loss	10(71.43)	1(7.14)

AEs were graded independently by two investigators per CTCAE v5.0; disagreements were resolved by third-party adjudication.Causality used three categories (chemotherapy-consistent, immune-consistent, indeterminate) with blinded adjudication per CTCAE v5.0; no immune-consistent colitis was observed (40).

### 3.4 The association between tumor markers and survival outcomes

**Figure 3:**
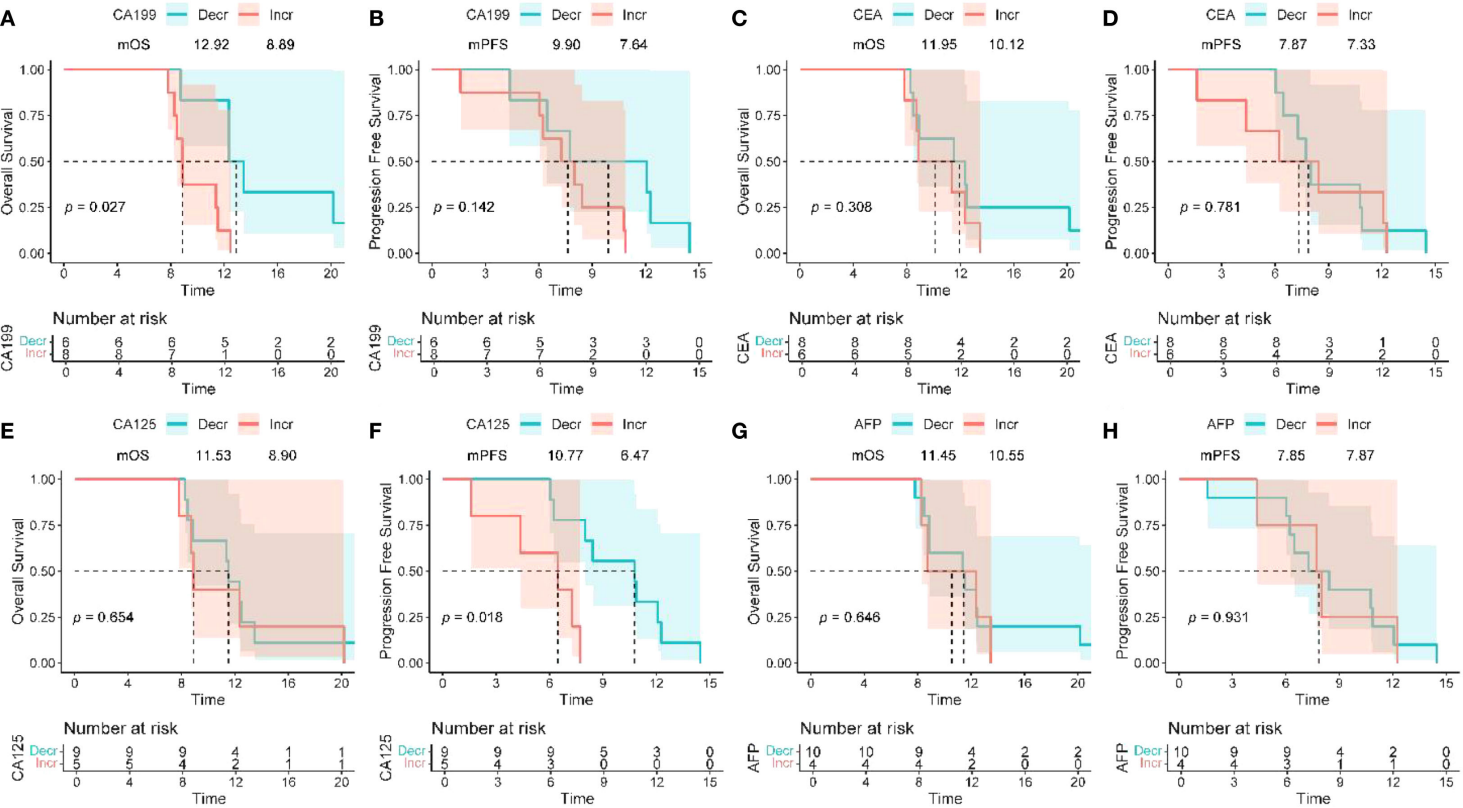
Association between peripheral blood biomarkers and treatment response. **(A, B)** The Kaplan–Meier curves of OS and PFS of patients stratified by CA19–9 change between baseline and optimal efficacy assessment time (decline vs. increase). **(C, D)** The Kaplan–Meier curves of OS and PFS of patients stratified by CEA change between baseline and optimal efficacy assessment time (decline vs. increase). **(E, F)** The Kaplan–Meier curves of OS and PFS of patients stratified by CA125 change between baseline and optimal efficacy assessment time (decline vs. increase). **(G, H)** The Kaplan–Meier curves of OS and PFS of patients stratified by CA125 change between baseline and optimal efficacy assessment time (decline vs. increase). OS, overall survival; PFS, progression - free survival; CA19 - 9, carbohydrate antigen 19 - 9; CEA, carcinoembryonic antigen; CA125, cancer antigen 125.

The dynamic changes in CA19–9 levels are significantly associated with survival outcomes: the median OS for patients with decreased CA19–9 after treatment is 12.92 months, significantly better than the non-compliant group (8.89 months) (HR = 4.77, 95%CI 1.20-19.01, P = 0.027) (**Figures 3A, B**); there is no significant difference in PFS between the two groups (P = 0.142). For CA125: patients with decreased CA125 after treatment had an extended median PFS (10.77 vs 6.47 months, HR = 7.56, 95%CI 1.42-40.22, P = 0.018), but it is not related to OS (P = 0.654) (**Figures 3C, D**); for CEA/AFP: changes in levels are not significantly associated with OS/PFS (both P>0.05) (**Figures 3E-H**).

### 3.5 Prognostic value of baseline inflammatory markers

**Figure 4:**
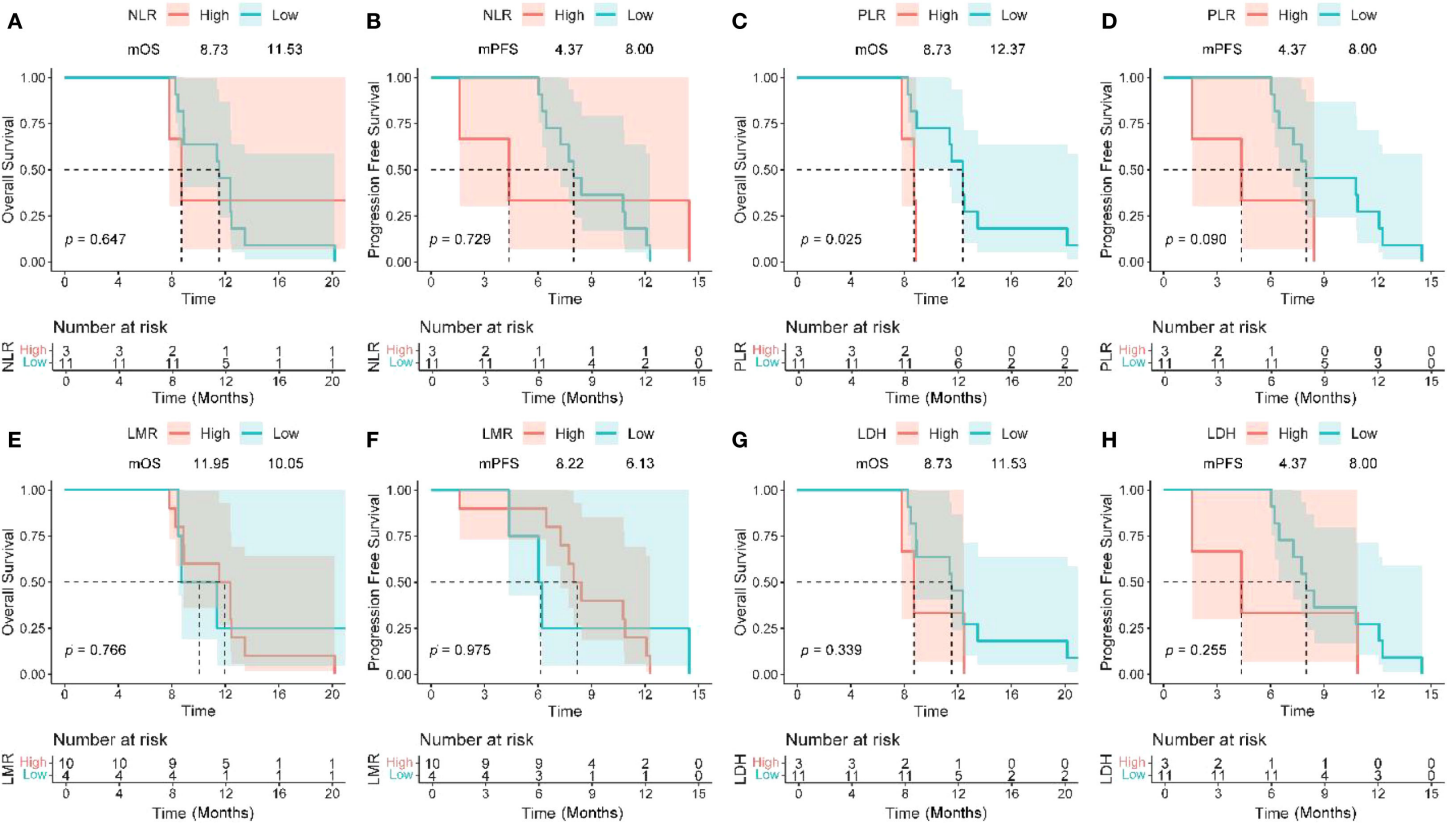
Kaplan–Meier analysis of survival and pretreatment inflammatory markers. OS and PFS based on NLR **(A, B)**, PLR **(C, D)**, LMR **(E, F)**, and LDH level **(G, H)**. OS, overall survival; PFS, progression-free survival; NLR, neutrophil-to-lymphocyte ratio; PLR, platelet-to-lymphocyte ratio; LMR, lymphocyte-to-monocyte ratio; LDH, lactate dehydrogenase; HR, hazard ratio; CI, confidence interval.

Patients with baseline PLR ≤ 165.62 had a median OS of 12.37 months compared to 8.73 months in the high PLR group (P = 0.025) (**Figures 4C, D**), while there was no significant difference in PFS between the two groups (P = 0.090). **Figures 4A–H** collectively show Kaplan–Meier curves of overall and progression-free survival according to pretreatment inflammatory indices (NLR, PLR, LMR, and LDH). Among these, only PLR was significantly associated with OS (P = 0.025), whereas the effects of NLR, LMR, and LDH were not statistically significant (all P > 0.05). Patients with LMR < 2.26 exhibited a higher objective response rate (ORR), but the difference did not reach statistical significance (P = 0.066). NLR ≤ 3.03, LDH ≤ 257 U/L, and PLR ≤ 165.63 were significantly associated with an increased disease control rate (DCR) (all P < 0.05) (**Table 6**).

**Table 6:** Statistical associations between blood indicators and tumor response.

Variable	N	PD N = 2	PR N = 2	SD N = 10	ORR N = 2	p-value	BH.p	DCR N = 12	p-value	BH.p
NLR	14					>0.99	>0.99		0.033	0.044
High		2.0 (100.0%)	0.0 (0.0%)	1.0 (10.0%)	0.0 (0.0%)			1.0 (8.3%)		
Low		0.0 (0.0%)	2.0 (100.0%)	9.0 (90.0%)	2.0 (100.0%)			11.0 (91.7%)		
PLR	14					>0.99	>0.99		0.033	0.044
High		2.0 (100.0%)	0.0 (0.0%)	1.0 (10.0%)	0.0 (0.0%)			1.0 (8.3%)		
Low		0.0 (0.0%)	2.0 (100.0%)	9.0 (90.0%)	2.0 (100.0%)			11.0 (91.7%)		
LMR	14					0.066	0.264		0.51	0.051
High		1.0 (50.0%)	0.0 (0.0%)	9.0 (90.0%)	0.0 (0.0%)			9.0 (75.0%)		
Low		1.0 (50.0%)	2.0 (100.0%)	1.0 (10.0%)	2.0 (100.0%)			3.0 (25.0%)		
LDH	14					>0.99	>0.99		0.033	0.044
High		2.0 (100.0%)	0.0 (0.0%)	1.0 (10.0%)	0.0 (0.0%)			1.0 (8.3%)		
Low		0.0 (0.0%)	2.0 (100.0%)	9.0 (90.0%)	2.0 (100.0%)			11.0 (91.7%)		

BH.p: Benjamini-Hochberg corrected p-values for multiple comparisons.

## 4 Discussion

The highly immunosuppressive microenvironment of pancreatic ductal adenocarcinoma (PDAC) (dense fibrous stroma, regulatory T cell infiltration) results in minimal efficacy of traditional immune checkpoint inhibitors (ICIs) (response rate of less than 2%) (6).This study employs Cadonilimab (a PD-1/CTLA-4 bispecific antibody) in combination with AG chemotherapy, achieving synergistic effects through dual mechanisms: the tetravalent structure of Cadonilimab specifically binds to PD-1/CTLA-4, blocking both immune checkpoint pathways, promoting local T cell activation in the tumor, while the Fc silent mutation reduces peripheral immunotoxicity (18); AG chemotherapy induces immunogenic cell death in tumor cells, releasing tumor-associated antigens and damage-associated molecular patterns, enhancing the antigen presentation function of dendritic cells (8). This “chemotherapy sensitization - immune activation” combination strategy provides a new pathway to overcome the inherent resistance of PDAC.

Research shows that the disease control rate (DCR) of combination therapy reaches 85.7% (12/14), significantly better than the traditional AG regimen’s 65%-70% (2). Although the objective response rate (ORR) is 14.3%, the improvement in DCR is directly associated with survival benefits — the median progression-free survival (mPFS) is 7.87 months, and the median overall survival (mOS) is 11.45 months, extending 0.9 months and 3.0 months respectively compared to the AG monotherapy (2). The survival outcomes (mOS 11.45 months) should be interpreted in the context of consistent patient characteristics with historical cohorts:Von Hoff et al. (2013) (2) reported 68% of patients with ECOG PS 0-1, consistent with our cohort’s functional status distribution and metastatic disease proportion (85.7%) align closely with Von Hoff et al. (2013) (2) and Conroy et al. (2011) (3). This minimizes bias from baseline differences, strengthening cross-study comparability. Potential heterogeneities (e.g., race, comorbidities) warrant confirmation in larger head-to-head trials.The high fibrous stroma of PDAC may delay the morphological response of tumors to treatment, while DCR can more comprehensively reflect the clinical benefits of “disease stability + partial response.” Notably, the modest improvement in mPFS (7.87 vs. 6.9 months with AG monotherapy) carries clinical significance in advanced PDAC, where rapid disease progression often leads to severe symptoms (e.g., pain, obstruction). Even short-term disease control can alleviate symptoms and improve quality of life. The high DCR (85.7%)—markedly higher than the 65%-70% with AG alone (2)—further supports the clinical value of this regimen, as stable disease in this setting translates to meaningful symptom management and reduced need for emergency interventions.

In PDAC, dense fibrotic stroma and poor perfusion can blunt early tumor shrinkage on conventional imaging (34, 35), while immunotherapy may produce atypical or delayed responses (36). Under these conditions, DCR pragmatically captures stable disease plus responses, providing an estimation-focused signal in a small, retrospective cohort with limited follow-up; ORR, PFS and OS are retained as key secondary outcomes for prospective confirmation.

This study found that the baseline PLR ≤165.62 may be a potential predictive factor worthy of further validation.Patients with PLR ≤ 165.6 had a significantly prolonged median overall survival (mOS) of 12.4 months, which is a 41.7% improvement compared to the high PLR group (8.7 months) (P = 0.025), and this is highly consistent with the conclusions of a meta-analysis that included 17 cohorts (HR = 1.28, P<0.0001) (44). This phenomenon may stem from a dual mechanism: low PLR indicates that lymphocyte-mediated anti-tumor immunity is dominant, while the secretion of platelet-derived pro-tumor factors (such as VEGF, TGF-β) is reduced, collectively enhancing the immune activation effect of the dual antibodies (45–47). Additionally, patients with NLR ≤ 3.03 and LDH ≤ 257 U/L had a significantly increased disease control rate (DCR) (P<0.05) (40), indicating that a reduced systemic inflammatory burden can improve treatment response. It is noteworthy that although the association between LMR and objective response rate (ORR) did not reach statistical significance (P = 0.066), the trend still suggests that the monocyte/lymphocyte balance affects tumor-killing potential. These serological markers constitute a low-cost, dynamically monitored prognostic assessment system, especially the strong predictive value of PLR provides a practical tool for clinical screening of beneficiary populations. Compared to previous PD-1 monoclonal antibody combined chemotherapy regimens (such as NLR for patient stratification) (9, 10), this study establishes the core role of PLR in PD-1/CTLA-4 bispecific antibody treatment for the first time, which may stem from the higher dependence of dual antibodies on the functional status of lymphocytes for synergistic blockade (18).

Patients with reduced CA19–9 levels after treatment showed significant survival benefits, with a median overall survival (mOS) of 12.92 months, extending 4.03 months compared to the non-target group (8.89 months) (HR = 4.77, 95% CI 1.20-19.01, P = 0.027, **Figures 3A, B**), suggesting that dynamic changes in CA19–9 may serve as a potential predictor of OS, requiring further validation. Notably, there was no statistically significant difference in progression-free survival (PFS) between the two groups (P = 0.142), indicating that CA19–9 is more suitable for assessing overall prognosis rather than short-term disease progression, which may be related to the high fibrous stroma in PDAC leading to delayed morphological responses of the tumor. This result is consistent with previous studies linking the decrease in CA19–9 levels to survival benefits (29, 48), further validating its clinical value in efficacy monitoring. Changes in CA125 levels were independently associated with prolonged PFS (P = 0.018) and may serve as a predictive factor for efficacy in specific treatment scenarios. Combined detection of biomarkers such as MUC5AC and DUPAN-2 can improve the limitations of false negatives of CA19–9 in Lewis-negative individuals (49), while the combined detection of CEA and CA125 aids in the differential diagnosis of pancreatic cancer and other gastrointestinal tumors (50, 51). Multi-biomarker dynamic monitoring provides precise assessment tools for personalized treatment.Exploratory analyses of other tumor markers (CEA, CA125, AFP) were conducted based on two considerations: first, their potential complementary value to CA19-9, as CA19–9 is unreliable in Lewis-negative patients (49), and CEA/CA125 have been reported to aid in differentiating pancreatic cancer from other gastrointestinal tumors (50, 51); second, preliminary evidence suggesting CA125 may correlate with PFS in some gastrointestinal malignancies (29, 51). However, it is important to clarify that these markers are not currently used in routine clinical practice for PDAC, and their predictive value in this setting requires further validation in larger cohorts.

In terms of safety, all patients experienced treatment-related adverse events (TRAE) of grade 1 or higher, with a grade 3 TRAE incidence of 57.1%, primarily consisting of nausea (21.4%) and appetite loss (14.3%), with no grade 4 or higher toxicity reported (**Table 5**). No immune-related adverse events (irAE) were reported in the entire group, which is significantly lower compared to previous PD-1/CTLA-4 dual-agent regimens (≥60% incidence of grade 3 or higher irAEs) (17), indicating that the Fc-silent design of Cadonilimab significantly reduces immune-related toxicity (18).

This study has the following limitations: First, as a single-center retrospective study, the sample size is small (n=14), and there is no control group, which may lead to selection bias, and the generalizability of the results needs to be validated; second, not all patients’ tumor tissues were obtained for molecular biological analysis, preventing exploration of the drug’s mechanisms of action and resistance at the genetic level;third, the prognostic analysis was conducted solely through serological markers and peripheral blood inflammatory indicators, lacking in-depth mechanism studies such as immune cell typing in the tumor microenvironment;additionally, the PLR cutoff (165.62) derived from limited data may carry overfitting risk; lack of tumor tissue molecular analysis and tumor microenvironment studies limits mechanistic insights; finally, the study did not explore the optimal dosage and treatment duration for combination therapy, which may affect the precision of efficacy assessment. We fully recognize the limitations of a single-center retrospective study with a small cohort (n=14). As shown in **Supplementary Table 1**, the study has limited statistical power: only large survival effects (HR ≈0.4–0.45) could be detected with 80% power (37, 38), whereas our observed HR ≈0.74 falls outside this range, indicating a non-trivial risk of type II error. The wide confidence intervals around DCR (≈60–96%) further highlight imprecision (39). These findings should therefore be interpreted as hypothesis-generating rather than confirmatory. In this context, our pilot study primarily aimed to observe safety signals and feasibility, while preliminary efficacy observations serve to inform the design of ongoing larger-scale, prospective studies (ChiCTR2400093744) that can provide more definitive evidence.These limitations suggest that the results of this study need to be further validated in larger sample size multi-center prospective studies.Our institution treated patients with advanced PDAC using AG monotherapy during the same period, but due to unobtained informed consent, these data were not included. A formal comparative analysis will be incorporated into our prospective trial (ChiCTR2400093744).

Baseline PLR ≤165.6 and on-treatment CA19–9 decline were prespecified for validation in our ongoing phase II trial (ChiCTR2400093744) with blinded independent analyses, and will be externally tested in a multicenter registry. Cut-off evaluation (time-dependent ROC with bootstrap validation), non-linear modeling (restricted cubic splines), and prespecified sensitivity analyses (handling of missing data, multiplicity, and alternative thresholds) were planned to ensure robustness (52–55).

Notably, to address the limitations of small sample size and single-arm design, we have initiated a single-arm, open-label, single-center phase II clinical trial (Chinese Clinical Trial Registry: ChiCTR2400093744) in 2024, which aims to further evaluate the efficacy and safety of Cadonilimab combined with AG chemotherapy in unresectable locally advanced or metastatic PDAC. This trial has enrolled some patients and is actively recruiting, with results expected to validate the findings of the current retrospective study and lay the groundwork for subsequent multi-center prospective trials.

## 5 Conclusion

In summary,this study suggests potential clinical value of Cadonilimab combined with AG chemotherapy: the DCR reached 85.7%, mOS was 11.45 months, with efficacy close to the FOLFIRINOX regimen but better safety; a baseline PLR ≤ 165.62 and dynamic decline of CA19–9 may suggest potential survival benefits, warranting further validation; grade 3 TRAE was mainly gastrointestinal reactions, with no severe immune-related toxicities. This regimen provides a new direction for immunotherapy in PDAC. Subsequent multicenter phase III trials are needed to further clarify the molecular characteristics of the benefiting population.

## Data Availability

The original contributions presented in the study are included in the article/**Supplementary Material**. Further inquiries can be directed to the corresponding authors.
