# Supplementary material for: Efficacy and safety of cadonilimab combined with AG chemotherapy in patients with unresectable locally advanced or metastatic pancreatic ductal adenocarcinoma: a retrospective real-world study
Source: Front Immunol. 2025 Oct 9;16:1654425. doi: 10.3389/fimmu.2025.1654425 (PMC12546067; doi:10.3389/fimmu.2025.1654425)
Supplement: Supplementary file 1 [file Table1.docx]

| **Variable** | **ORR**  N = 2 | **Others**  N = 12 | **α**  **error** | **β**  **error** | **p-value** | **DCR**  N = 12 | **Others**  N = 2 | **α**  **error** | **β**  **error** | **p-value** |
| --- | --- | --- | --- | --- | --- | --- | --- | --- | --- | --- |
| **NLR** |  |  | 0.05 | 0.91 | >0.99 |  |  | 0.05 | 0.31 | 0.033 |
| High | 0.0  (0.0%) | 3.0 (25.0%) |  |  |  | 1.0 (8.3%) | 2.0 (100.0%) |  |  |  |
| Low | 2.0 (100.0%) | 9.0 (75.0%) |  |  |  | 11.0 (91.7%) | 0.0 (0.0%) |  |  |  |
| **PLR** |  |  | 0.05 | 0.91 | >0.99 |  |  | 0.05 | 0.31 | 0.033 |
| High | 0.0 (0.0%) | 3.0 (25.0%) |  |  |  | 1.0 (8.3%) | 2.0 (100.0%) |  |  |  |
| Low | 2.0 (100.0%) | 9.0 (75.0%) |  |  |  | 11.0 (91.7%) | 0.0 (0.0%) |  |  |  |
| **LMR** |  |  | 0.05 | 0.49 | 0.066 |  |  | 0.05 | 0.92 | 0.51 |
| High | 0.0 (0.0%) | 10.0 (83.3%) |  |  |  | 9.0 (75.0%) | 1.0 (50.0%) |  |  |  |
| Low | 2.0 (100.0%) | 2.0 (16.7%) |  |  |  | 3.0 (25.0%) | 1.0 (50.0%) |  |  |  |
| **LDH** |  |  | 0.05 | 0.91 | >0.99 |  |  | 0.05 | 0.311 | 0.033 |
| High | 0.0 (0.0%) | 3.0 (25.0%) |  |  |  | 1.0 (8.3%) | 2.0 (100.0%) |  |  |  |
| Low | 2.0 (100.0%) | 9.0 (75.0%) |  |  |  | 11.0 (91.7%) | 0.0 (0.0%) |  |  |  |

Notes:

- Type I error (α) was fixed at 0.05 according to study design.

- Type II error (β) was estimated post hoc based on the observed sample size (n=14) and event counts.

- For survival endpoints, calculations were performed using the Freedman/Schoenfeld approximation; for binary endpoints, 95% confidence intervals were estimated using the Wilson method.

- Due to the small sample size, statistical power was limited and β errors were generally high, underscoring the exploratory, hypothesis-generating nature of this analysis.
